# Psychological problems in general population during covid-19 pandemic in Pakistan: role of cognitive emotion regulation

**DOI:** 10.1080/07853890.2020.1853216

**Published:** 2020-12-16

**Authors:** Maryam Riaz, Mueen Abid, Zaqia Bano

**Affiliations:** Department of Psychology, University of Gujrat, Gujrat, Pakistan

**Keywords:** Covid-19 pandemic, cognitive emotion regulation, regression

## Abstract

**Objectives:**

To explore psychological problems (Anxiety, Depression and Stress) in general population during Covid-19 pandemic. To find predictive effects of cognitive emotion regulation on psychological problems.

**Methodology:**

Convenient sampling technique was used to obtain the sample of 500 participants (Male = 239, Female = 261). Research instrument consists of four parts. First part comprised of consent form, second part was about demographic profile, third part was Depression, Anxiety and Stress scale (DASS-21) while Cognitive Emotion Regulation Questionnaire was the last part of the instrument.

**Results:**

SPSS 23.0 (Statistical Package for Social Sciences) version was used for study analysis. Descriptive statistics used to summarize the raw data. The inferential statistics such as regression, correlation and t-test were used to calculate the findings according to research objectives. Results indicated that 33%, 40% and 27% individuals were experiencing Depression, Anxiety and Stress respectively during Covid-19 pandemic. Among these participants, 48% (*N* = 242) were experiencing normal level of all these targeted psychological problems while remaining 52% (*N* = 258) respondents have mild to very severe level of all these disorders. Furthermore, findings of linear regression analysis illustrated that cognitive emotion regulation significantly predicts psychological problems [*R*^2^=.216; *F* = 51.223, *p* < .01] and 21% variation in psychological problems is due to cognitive emotion regulation.

**Conclusion:**

This study recommended that policy makers must develop and implement some necessary programmes to prevent and cure people from devastating psychological and mental health consequences of covid-19 on priority basis.

## Introduction

Human civilization perhaps is facing one of the most critical juncture of this century which challenged its existence. In December 2019, Corona virus Disease (Covid-19), which is a new virus discovered with its outbreak in Wuhan, a main city of China and then spread all over the world rapidly. On 26 February 2020, first patient of Covid-19 appeared in Pakistan and after sometimes the situation became worse.

As this virus is new and World Health Organization [1], on 11 March 2020 has declared it a pandemic, there is a great need to explore various aspects and effects of this universal problem. In this regard, most of the research work focussed on identifying the epidemiology, medical description, genomic characterization, clinical characteristics, mode of transmission, incubation period, symptoms, contain the stretch of the virus, governance, economical issues and mainly on treatment of this new virus and disease [2–4]. It is quite reasonable that there is a great need to understand the new virus biologically and medically but at the same time the psychological and psychiatric requirements cannot be ignored or overlooked at any phase of pandemic control and management [5–7]. That is why enormous psychological researches have been conducted all over the world to uncover psychological impact of the novel disease on individuals. Santamaría et al. [8] indicated that health professionals who are front line soldiers were experiencing a significant level of depression, anxiety, stress and insomnia and the higher level was prevailing among female health workers than male. Hao et al. [9] indicated that psychiatric patients were at higher risk to develop mental health problems as compared to general population whereas, Xiong et al. [10] systematically reviewed that general population in China, Italy, Spain, Iran, United States, Nepal, Denmark and Turkey is also extremely experiencing Depression (14.6% to 48.3%), Anxiety (6.33% to 50.9%), Stress (8.1% to 81.9%), Psychological Distress (34.43% to 38%) and post-traumatic stress disorder (7% to 53.8%) during covid-19. Tee et al. [11] indicated that 17%, 29%, 13% respondent were experiencing depression, anxiety and stress respectively in Philippine during first month of pandemic. Furthermore, it has been indicated that at the initial stage of covid-19 alert, symptoms of depression, anxiety and stress were generally low but Stay-At-Home orders increased the severity level of problems. Younger respondents and people with chronic illnesses were at greater risk than other groups [12,13]. The scientometric analysis indicated that psychological research is one of the top 10 research fields during COVID-19 pandemic and most of the researches are mainly contributed by China, United states and European countries, so there is a great need to explore the phenomena in low or middle income states to better understand the situation [14]. Condition in Europe and Asia is different during pandemic as indicated by Wang et al. [15–17] that people in Poland were facing more level of depression, anxiety and stress than Chinese. To fill this gap of research, present study was planned to check psychological problems prevail in general population in Pakistan because identification of problems lead towards its solution. Under the umbrella of psychological problems, depression, anxiety and stress were targeted. Furthermore role of different demographic variables was also explored. There are few reasons to select this topic and population for study. Mainly, Pakistan is the fifth most populous country in the world with poor economical conditions. Basic family system is joint family in which generations are living together even in small houses. In this scenario importance of social distancing and being unemployed imposed a challenged to the mental health of the people along with their physical fitness. According to one study individuals are going through the fear of death, fear of being infected, depression, anxiety, anger and other mental health problems during outbreak of this pandemic [18].

It has been identified that there are few factors which play considerable function in the emergence, maintenance and even for the management of the psychiatric morbidity like coping styles and social support [19]. Sensory processing patterns (sensory/avoiding, low registration, sensation seeking) and history of moderate-severe childhood maltreatment also predicts mental health problems [20,21]. While experiencing traumatic situations, general population behave differently [22]. The individuals who failed to cope positively could develop psychological issues or mental health problems like post-traumatic stress disorder (PTSD), substance abuse, anxiety and mood disorders. Many researchers have identified that how cognitive regulation of emotions played significant role in emergence of many psychological problems among individuals [23–26]. The main model which explained the role of nervous system in controlling emotions is Cognitive emotion regulation strategies model. This model discusses how different cognitive processes play role to effectively handle emotional arousal [23,27]. There are nine theoretically identified strategies of cognitive emotion regulation: self-blame, rumination, other-blame, catastrophizing, positive refocusing, putting into perspective, positive reappraisal, planning and acceptance [28].

In this context, unpredictable and unexpected disaster like COVID-19 is the certain situation which can trigger intense emotional arousal and demand for emotional control and regulation is substantial [29]. Moreover, these disasters may elicit extreme amount of negative emotions like hopelessness, horror and fear. If extreme negative emotion arousal is not regulated effectively, it can produce long lasting psychiatric illnesses [30,31]. For this reason present study aimed to investigate the predictive effects of cognitive emotion regulation on development of targeted psychological problems at the time of covid-19 spread so that individuals who are suffering from psychological issues and deficient in positive cognitive regulation of emotions could be identified and undergo timely treatment and intervention. Recently, cognitive emotion regulation has become core process to manage psychosomatic illnesses and psychiatric disorders. Ho et al. [32] indicated that some mental health strategies such as psycho education, Cognitive Behaviour Therapy and mindfulness-based therapy play significant role to prevent and treat mental health problems during covid-19 pandemic. Cognitive Behaviour Therapy can change maladaptive coping styles such as avoidance, confrontation, aggression and self blame into adoptive coping.

**Figure 1.**
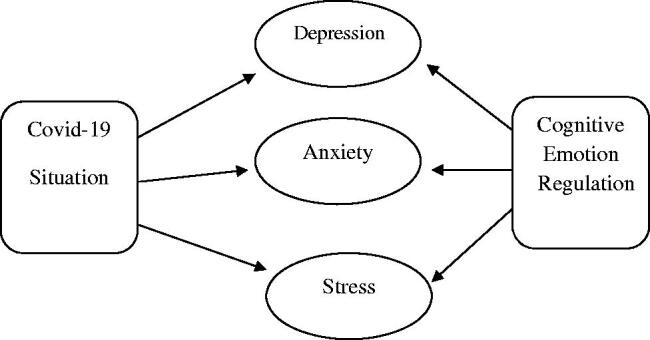
illustrated the hypothetical model of present research.

Collectively, present study was conducted with two main objectives. Firstly, to identify how covid-19 pandemic develop Depression, Anxiety and Stress among general population and then how cognitive emotion regulation predicts these targeted psychological issues (Figure 1).

## Objectives of the study

The study was conducted with four subsequent objectives such as to explore psychological problems (Depression, Anxiety and Stress) in general population, to find cognitive emotion regulation as a predictor of psychological problems in general population, to investigate cognitive emotion regulation as a predictor of sub domains of psychological problems (Depression, Anxiety and Stress) in general population and to identify the association of significant demographic variables with cognitive emotion regulation and targeted psychological problems.

## Methodology

### Participants and data collection

Sample was drawn with the help of convenient sampling technique because it was extremely difficult to use probability sampling method due to lockdown. A total of 500 respondents with different demographic characteristics participated in the research process.

#### Inclusion and exclusion criteria

People with age limit of 12 years and above living in district Gujrat were included while participants below 12 years of age and from other districts of Pakistan were excluded from the study. Furthermore, participants with prior history of psychiatric morbidity and chronic physical diseases such as cancer and heart diseases were also excluded from the research.

Participants were approached conveniently at their homes with Standard Operating Procedures (SOPs) related to covid-19. Researchers had to convinced people to participate in the study due to its significance at the time of covid-19 spread. The following research instruments were given to the participants after elaborating the objectives and nature of the study. Privacy and confidentiality ensured to the respondents. Their participation was voluntary and they could skip research process any time. At the end of the data collection participants were acknowledged for their valuable contribution in research process.

### Research instruments

#### Consent form

Consent form contained information related to the researcher and research objectives. Willing participants signed the consent sheet and participated in research process.

#### Demographic sheet

Next part of study tool was about demographic profile like gender, age, level of education, current marital position, family structure, employment status, family income and residential area (Table 1).

**Table 1.** Demographic profile of respondents (*N* = 500).

Variables	N	%
Age		
12–19	119	24
20–30	152	30
31–45	126	25
46–55	83	17
56–Above	20	4
Gender		
Male	239	48
Female	261	52
Family system		
Nuclear	136	27
Joint	364	73
Education		
Ill-Literate	73	15
Primary	24	5
Elementary	12	2
Matriculation	81	16
F.A/F.Sc	59	12
B.A/B.Sc	62	12
M.A/M.Sc	64	13
BS(Honors)	104	21
MPhil	15	3
PhD	6	1
Employment status		
Employed	289	57
Unemployed	211	42
Marital status		
Married	237	47
Unmarried	172	34
Separation	40	8
Widow	36	7
Divorced	15	3
Family Income		
Less than 20000	79	16
20000–40000	228	46
41000–60000	106	21
Above 60000	87	17
Residential area		
Urban	228	46
Rural	272	54

#### Depression, anxiety and stress scale (DASS-21)

Additionally, Depression, Anxiety and Stress scale (DASS-21), translated into Urdu language was used to assess psychological problems [33]. It is self report scale with three different categories: Depression, Stress and Anxiety and each category contain seven items. It is four point liker scale ranges from 0 to 3. Cut-off scores greater than 7, 9 and 14 for anxiety, depression and stress respectively, indicate existence of the problem. The DASS-21 demonstrated excellent reliability as the depression, anxiety and stress subscales had 0.81, 0.89 and 0.78 Cronbach’s alpha values respectively [34]. DASS-21 has been used by many researches during covid-19 [9,15–17,35–37].

#### Cognitive emotion regulation questionnaire

Last part of the research instrument was cognitive emotion regulation scale with Urdu translation [38]. This questionnaire is consists of 36 items, categorize into nine conceptually diverse subscales and each subscale comprises of 4 items. The scale is on 5-point Likert format. Research exhibits that subscales encompass excellent internal consistencies ranges from 0.68 to 0.86 [39].

### Data analysis

Statistical Package for Social Sciences, version 23.0 was used for research analysis. Descriptive statistics helped to summarize the data while inferential statistics such as regression, correlation and *t*-test were used to calculate the findings according to research objectives. To be considered significant p value should be equal to or less than .05.

## Results

Table 2 identifies the reliability analysis of variables used in the study. Both scales of the research have high reliability which confirmed that these tools are appropriate for this research. The cognitive emotion regulation questionnaire has reliability with alpha value of 0.762 while Depression, Anxiety and Stress scale has Cronbach Alpha value of 0.741.

**Table 2.** Cronbach Alpha of Scales of the Study (*N* = 500).

	Total items	Cronbach alpha
Cognitive Emotion Regulation Questionnaire	36	0.762
Depression, Anxiety and Stress Scale	21	0.741

Table 3 indicates the intercorrelation among cognitive emotion regulation and targeted psychological problems (Depression, Anxiety and Stress). Above correlation matrix illustrated that all study constructs are significantly correlated.

**Table 3.** Intercorrelation between Cognitive Emotion Regulation and Sub domains of psychological problems.

	CER	Depression	Anxiety	Stress	
CER	1				
Depression	−0.472**	1			
Anxiety	−0.138**	0.286**	1		
Stress	−0.144**	0.380**	0.835**	1	

Note: ** *p*<.01; **p<.05.

Table 4 indicated that depression, anxiety and stress have the percentages of 33 (*N* = 163), 40 (*N* = 201) and 27 (*N* = 136) respectively. Under the umbrella of psychological problems, the score of anxiety is higher than other problems like depression and stress. So anxiety is most prevalent psychological problem followed by depression and stress during Covid-19 in general population. Furthermore, 16% (*N* = 81) participants have normal depression while 12% (*N* = 59) are experiencing mild to moderate level of it. In anxiety, 97(19%) respondents have normal level while mild and moderate category represented 09% (*N* = 44) participants. Findings further illustrated that number of respondents with severe and very severe anxiety level is also critical (*N* = 60, %=12). Moreover, 60 (12%) participants are going through mild and moderate level of stress while 02% (*N* = 12) experience severe and very severe stress.

**Table 4.** Frequencies and percentages of sub domains of psychological problems with severity (*N* = 500).

Variables	Categories	F	%
Depression score		163	33
	Normal	81	16
	Mild and moderate	59	12
	Severe and very severe	23	05
Anxiety Score		201	40
	Normal	97	19
	Mild and moderate	44	09
	Severe and very severe	60	12
Stress Score		136	27
	Normal	64	13
	Mild and moderate	60	12
	Severe and very severe	12	02

Linear regression analysis carried out to investigate predictive effects of cognitive emotion regulation on psychological problems. Findings indicated, cognitive emotion regulation as a significant predictor of psychological problems among general population [R^2^=0.216; *F* = 51.223, *p*<.01]. Additionally, Table 5 depicts that 21% variation in psychological problems is due to different strategies of cognitive emotion regulation.

**Table 5.** Summary of Regression Analysis of cognitive emotion regulation as the predictor of psychological problems (*N* = 500).

Variables	R	R^2^	Adjusted R^2^	F	*p* Value
CER Psychological problems	0.452	0.216	0.212	51.223	.000

CER: Cognitive Emotion Regulation.

Table 6 illustrated that cognitive emotion regulation significantly predicted all domains of psychological problems. Findings of regression analysis indicated that cognitive emotion regulation can cause depression [R^2^=0.379, *F* = 119.731, *p*<.01] with 37.9% explained variance. Furthermore, cognitive emotion regulation significantly predicted anxiety in general population with 43.2% explained variance. Stress is also significantly predicted by cognitive emotion regulation [R^2^=0.307, *F* = 87.145, *p*<.01]. Findings demonstrated that 30.7% variance in stress score is because of cognitive emotion regulation.

**Table 6.** Summary of Regression Analysis of cognitive emotion regulation as the predictor of sub domains (Depression, Anxiety and Stress) of psychological problems (*N* = 500).

Variables	R	R^2^	Adjusted R^2^	F	*p* Value
CER Depression	0.615	0.379	0.384	119.731	.000
CER Anxiety	0.644	0.432	0.398	133.225	.000
CER Stress	0.552	0.307	0.303	87.145	.000

Table 7 showed findings of correlation analysis among cognitive emotion regulation, psychological problems and education in general population. Findings illustrated that these variable have significant relationship. Education of participants correlated positively with cognitive emotion regulation and has negative correlation with psychological problems.

**Table 7.** Bivariate Correlation between cognitive emotion regulation, psychological problems and education (*N* = 500).

	Variable	Cognitive emotion regulation	Psychological problems	*p* Value
	Education	0.321**	−0.725**	.000

**Correlation is significant at the .01 level (2-tailed).

Table 8 indicated gender differences on Cognitive Emotion Regulation and psychological problems in participants. Both groups are significantly different on targeted variables. Mean score indicated that male participants score higher (*M* = 24.2, SD = 4.6) on cognitive emotion regulation than female (*M* = 17.3, SD = 3.6) while in psychological problems domain female participants have higher scores (*M* = 53.7, SD = 14.9) than their counterpart (*M* = 40.1, SD = 10.8).

**Table 8.** Mean, standard deviation and t-value of male and female on cognitive emotion regulation and psychological problems (*N* = 500).

	Male (*N* = 239)		Female (*N* = 261)				95 % CI
Measures	M	S.D	M	S.D	t-value	*p* Value	Lower limit to upper limit
Cognitive emotion regulation	24.2	4.6	17.3	3.6	2.5	.01	−2.21 to .248
Psychological problems	40.1	10.8	53.7	14.9	2.37	.02	−8.87 to 2.09

**p<.05.

## Discussion

Findings of present research are discussed below according to its objectives.

The first purpose of the research was to identify psychological problems in general population during covid-19. Findings revealed that 33% participants have depression, 40% are with anxiety and 27% have stress problem. Furthermore, 48% individuals (*N* = 242) were experiencing normal level of all these targeted psychological problems while remaining 52% (*N* = 258) respondents have mild to very severe level of all these disorders. These findings are in lined with different researches conducted during covid-19 and before. A study carried out in China at time of this pandemic spread demonstrated that people are suffering a lot during this pandemic. Additionally, in China, more than half of the study respondents had considerable mental health consequences of this traumatic outbreak [15–17]. Another research from Denmark also found psychological consequences of covid-19 on people and reported that this pandemic negatively effects psychological wellbeing and creates depression [40]. Moreover, a survey carried out by American Psychiatric Association in United States also indicated that nearly half of the participants have psychological problems like anxiety during covid-19 [41].

The second objective of this research was to explore cognitive emotion regulation as the predictor of psychological problems in general population. Results indicated that cognitive emotion regulation significantly predicts psychological issues in participants during traumatic situation like covid-19. Previous researches also confirmed this prediction. considerable researches have been conducted to investigate the predictive effects and relationship between different coping strategies such as cognitive emotion regulation and psychological problems like depression, drug abuse, anxiety, masochism, stress after accident, borderline personality disorder and eating disorder [23–27,42].

To find predictive effects of cognitive emotion regulation on sub domains of psychological problems like depression, stress and anxiety was third objective of research. Results of regression analysis demonstrated statistically significant predictive effects of cognitive emotion regulation on depression, stress and anxiety in general population at the time of covid-19 spread. Considerable amount of researches indicated that the cognitive emotion regulation strategies used by individuals influence depression development. Failure to adopt appropriate strategies during traumatic or difficult situations can lead to depressive disorders [24,42,43]. Furthermore, a research carried out by Ongen [25] in Turkey also indicated that different strategies of cognitive emotion regulation significantly predict depression among individuals. Similarly, earlier studies also found predictive effects of Cognitive Emotion Regulation on anxiety. The way individuals adopts particular cognitive emotion regulation strategy to effectively handle problematic situation also affects their level of anxiety [44]. Furthermore, several clinical studies have demonstrated that the ineffective or inappropriate cognitive regulation of emotions is a vital element in development, maintenance and management of psychological disorders like anxiety and depression [45–47]. Correspondingly, stress is also caused by inappropriate practice of cognitive emotion regulation as indicated by a study conducted in Iran by Oftadehal et al. [48] to find predictive relationship of stress and cognitive emotion regulation. This study suggested that different cognitive emotion regulation strategies strongly predict stress in participants. Another research carried out by Solgi and Yaseminejad [49] identified that some cognitive emotion regulation strategies like self-blame, low positive reappraisal and rumination have high potential to predict stress among people.

Last research objective was to investigate association among demographical variables, cognitive emotion regulation and psychological problems. Findings of research identified a significant positive correlation among level of education, cognitive emotion regulation and psychological problems. There are many researches which have found that higher level of education increase skills of people, improve cognition and afford better coping strategies and all of these things ultimately improve psychological health of individuals [50,51]. Furthermore, significant differences in gender were explored in present research among cognitive emotion regulation and psychological problems which are confirmed by several previous studies which suggested that such differences occurred due to the way both gender respond to negative events in life and try to manage their psychological problems [39,52,53].

## Conclusion

Present study concluded that 72% participants are experiencing any of the targeted psychological issue during covid-19 and cognitive emotion regulation significantly predicts depression, anxiety and stress. These findings demonstrate the need of psychological planning, interventions and therapeutic programs such as workshops at community and educational institutions to create awareness, self management skills training to deal with traumatic situations effectively, counselling, cognitive behaviour therapy to change maladaptive cognitive and emotional dealing into positive ones and mindfulness based training to prevent and cure these mental health issues to create a fully functioning society before it is too late.
